# Phytochemical Profile and Biological Activities of Crude and Purified *Leonurus cardiaca* Extracts

**DOI:** 10.3390/plants10020195

**Published:** 2021-01-21

**Authors:** Simone Angeloni, Eleonora Spinozzi, Filippo Maggi, Gianni Sagratini, Giovanni Caprioli, Germana Borsetta, Gunes Ak, Kouadio Ibrahime Sinan, Gokhan Zengin, Sabrina Arpini, Giacomo Mombelli, Massimo Ricciutelli

**Affiliations:** 1 School of Pharmacy, University of Camerino, via Sant’Agostino 1, I-62032 Camerino, Italy; simone.angeloni@unicam.it (S.A.); eleonora.spinozzi@unicam.it (E.S.); gianni.sagratini@unicam.it (G.S.); giovanni.caprioli@unicam.it (G.C.); germana.borsetta@unicam.it (G.B.); massimo.ricciutelli@unicam.it (M.R.); 2 International Hub for Coffee Research and Innovation, I-62020 Belforte del Chienti, Italy; 3 Physiology and Biochemistry Laboratory, Department of Biology, Science Faculty, Selcuk University, Konya 42130, Turkey; akguneselcuk@gmail.com (G.A.); sinankouadio@gmail.com (K.I.S.); gokhanzengin@selcuk.edu.tr (G.Z.); 4 Indena SpA, I-20049 Settala, Italy; sabrina.arpini@indena.com (S.A.); giacomo.mombelli@indena.com (G.M.)

**Keywords:** *Leonurus cardiaca*, antioxidants, enzyme inhibitory properties, HPLC-MS, plant extracts, polyphenols

## Abstract

*Leonurus cardiaca* L. (Lamiaceae) is a perennial herb distributed in Asia and Southeastern Europe and has been used in traditional medicine since antiquity for its role against cardiac and gynecological disorders. The polar extracts obtained from *L. cardiaca* aerial parts contain several compounds among which alkaloids, iridoids, labdane diterpenes, and phenylethanoid glycosides play a major role in conferring protection against the aforementioned diseases. On the other hand, the antioxidant activities and the enzyme inhibitory properties of these extracts have not yet been deeply studied. On the above, in the present study, crude and purified extracts were prepared from the aerial parts of *L. cardiaca* and have been chemically characterized by spectrophotometric assays and HPLC-DAD-MS analyses. Notably, the content of twelve secondary metabolites, namely phenolic acids (chlorogenic, caffeic, caffeoylmalic and *trans*-ferulic acids), flavonoids (rutin and quercetin), phenylethanoid glycosides (verbascoside and lavandulifolioside), guanidine pseudoalkaloids (leonurine), iridoids (harpagide), diterpenes (forskolin), and triterpenes (ursolic acid), has been determined. Furthermore, the extracts were tested for their antioxidant capabilities (phosphomolybdenum, DPPH, ABTS, FRAP, CUPRAC, and ferrous chelating assays) and enzyme inhibitory properties against cholinesterase, tyrosinase, amylase, and glucosidase. The purified extracts contained higher phytochemical content than the crude ones, with caffeoylmalic acid and verbascoside as the most abundant compounds. A linear correlation between total phenolics, radical scavenging activity, and reducing power of extracts has been found. Notably, quercetin, caffeic acid, lavandulifolioside, verbascoside, chlorogenic acid, rutin, and ursolic acid influenced the main variations in the bioactivities found in *L. cardiaca* extracts. Our findings provide further insights into the chemico-biological traits of *L. cardiaca* and a scientific basis for the development of nutraceuticals and food supplements.

## 1. Introduction

*Leonurus cardiaca* L., also known as motherworth, Echte Herzgespann, and agripaume, is a perennial herb belonging to the Lamiaceae family and native to Asia and Southeastern Europe. This plant can reach a height of 1 m and its leaves are palmately lobed and covered by stiff hairs. The flowers are pink, around 1 cm long, and grouped in 10–20 clusters [1]. *L. cardiaca* has played an important role in traditional medicine as a remedy against heart failure, tachyarrhythmia, and several other cardiac diseases [2], being also described in 15th and 16th century European herbals as “Gart der Gesundheit” [3]. Other known therapeutic applications include the treatment of neurological, cardiac and gynecological disorders, and thyroid dysfunctions [4,5]. Scientific evidence supports its antioxidant, antimicrobial, and anti-inflammatory properties, as well as its beneficial effects against digestive disorders and bronchial asthma [6]. 

Different classes of secondary metabolites have been identified in *L. cardiaca*. In particular, flavonoids (e.g., flavonols such as quercetin and rutin, flavones such as genkwanin and apigenin, and their relative glycosides) [7], phenylethanoid glycosides (e.g., verbascoside and lavandulifolioside) [8], iridoids (e.g., harpagide), labdane diterpenes (e.g., forskolin) [9,10], *p*-hydroxycinnamic acid derivatives (e.g., ferulic, chlorogenic and caffeic acids) [11], guanidine pseudoalkaloids (e.g., leonurine) [12], and betains (e.g., stachydrine, and turicin) [10]. The essential oil obtained from the leaves was reported to contain *β*-caryophyllene and *α*-humulene as the major compounds. For its various biological activities, *L. cardiaca* has been included in the European Pharmacopoeia since 1941, where it is indicated as an ingredient for homeopathic and phytopharmaceutical preparations [13]. Extracts obtained from this plant are also recommended by the European Medicines Agency (EMA) as potential drugs [5]. 

The pharmacological potential of plants traditionally used as herbal remedies is gaining more and more attention by actual research, in order to develop not only new drug candidates but also nutraceutical products and food supplements. In this framework, it is of pivotal importance to extensively characterize plant extracts in order to increase the knowledge of their content of marker compounds and their relationship with biological activities. This is particularly helpful during the development of food supplements and nutraceutical products. Among several illnesses, aging-related diseases are becoming the new challenge of the 21st century. Oxidative stress plays a crucial role in the development and progression of these diseases, including arthritis, diabetes, dementia, cancer, atherosclerosis, vascular diseases, obesity, osteoporosis, and metabolic syndromes. Antioxidant compounds such as polyphenols, vitamins, carotenoids, and others are involved in a broad spectrum of antioxidant processes and exert a protective role in the pathogenesis of age-related diseases [14]. In addition, several approaches have been employed for the treatment of chronic diseases such as diabetes and Alzheimer’s disease, among which the inhibition of target enzymes is one of the most used to alleviate the symptoms associated with these illnesses. In this regard, some enzymes, such as cholinesterase, amylase, glucosidase, tyrosinase, and lipase, are considered important pharmaceutical targets [15,16]. Nevertheless, a few studies have hitherto focused on the enzyme inhibitory properties of *L. cardiaca* extracts, whereas most of them have focused on the properties of single isolated compounds [17,18,19]. Moreover, the antioxidant activities of *L. cardiaca* extracts have been evaluated only for a limited number of in vitro mechanisms [20,21]. To the best of our knowledge, no reports are available in the literature on the chemical and biological characterization of *L. cardiaca* extracts subjected to the purification process. The latter is very important in the pharmaceutical and nutraceutical industry in order to remove non-useful macromolecules and sugars and to enrich the bioactive fraction [22].

On the above, the objective of the present study was the development of a High-Performance Liquid Chromatography coupled to Diode Array Detector and Mass Spectrometry (HPLC-DAD-MS) analytical method for the quantitative evaluation of the main bioactive compounds of *L. cardiaca* crude and purified extracts belonging to different chemical classes, such as phenolic acids, flavonoids, phenylethanoid glycosides, guanidine pseudoalkaloids, iridoids, labdane diterpenes, and triterpenes. The total phenolic content (TPC) and total flavonoid content (TFC) have also been determined. Moreover, the antioxidant and enzyme inhibitory properties were evaluated. In particular, the antioxidant activity was assessed through different antioxidant mechanisms, relying on several methods such as phosphomolybdenum, antiradical (DPPH and ABTS), reducing power (FRAP and CUPRAC), and ferrous chelating assays. On the other hand, the inhibitory activity was tested on different enzymes: acetylcholinesterase (ACh, EC3.1.1.7), butyrylcholinesterase (BuCh, EC 3.1.1.8), tyrosinase (1.14.18.1), *α*-glucosidase (EC 3.2.1.20), and *α*-amylase. Finally, statistical analyses such as Pearson’s correlation and partial least square analysis were applied, respectively, to study the relationship between TPC, TFC, and the biological activities and to evaluate the contribution of the various phytochemicals to the biological activities.

## 2. Results and Discussion

### 2.1. Optimization and Validation of HPLC-DAD-MS Analytical Method

The quantitative analysis of twelve compounds, including guanidine pseudoalkaloids, flavonoids, iridoids, phenylethanoid glycosides, phenolic acids, and triterpenes, in different extracts of *L. cardiaca* has been achieved using an HPLC-DAD-MS system. Ion species and MS parameters of each analyte were selected and optimized by individually injecting each standard solution (1 µL, 10 mg L^−1^) in flow injection analysis (FIA). Studying the obtained mass spectra, the most abundant ion was used for quantification while other ions were used to confirm the analyte structure. The ions selected for quantitation were [M-H]^−^ ion species for all monitored compounds, except for harpagide, which was [M+HCOO]^−^, giving the 409 *m*/*z* ion. The ion species of lavandulifolioside were selected using literature data [23] and by injecting a *L. cardiaca* extract in SCAN mode (100–1000 *m*/*z*). The peak at retention time of 11 min was assigned to lavandulifolioside since the mass spectrum was essentially characterized by the [M-H]^−^ ion (755 *m*/*z*) and [M+Cl]^−^ species (791 *m*/*z*), the latter having a lower abundance. DAD analysis confirmed the lavandulifolioside occurrence at Rt of 11 min. The 775 *m*/*z* ion was chosen for the quantitative determination, whereas the calibration curve of verbascoside was used for lavandulifolioside quantitation as well. Several attempts were performed to optimize the analyte separation such as various mobile phases (water (A)/methanol (B), water (A)/methanol (B), both with 0.1% of formic acid) and different gradients. The best two gradients employed formic acid in both solvents of mobile phases and the first was as follows: 0 min, 40% B; 0–5 min, isocratic condition, 40% B; 5–20 min, 40–90% B; 20–30 min, isocratic condition, 90 B; 30–37 min, 40% B. The second gradient was chosen for the analysis since it determined good separation and satisfactory resolution; its description is given in the Materials and Methods section. 

The new analytical method was validated by studying various parameters such as linearity, limit of detection (LOD), limit of quantification (LOQ), and repeatability using two different detectors, i.e., DAD and MS (Table 1).

The linearity was evaluated by injecting seven different concentrations of standard analyte solutions and preparing the respective calibration curves. Good linearity was obtained using both detectors since the determination coefficients (R^2^) were in the range of 0.9908–0.9997 when the MS system was employed and in the range of 0.9952–0.9995 with the DAD. LOD and LOQ values were calculated by injecting gradually lower concentrations of standard mixtures and the concentration with signal-to-noise ratio (SNR) of 3 was assigned to LOD while that with SNR of 10 was attributed to LOQ. The acquisitions with the MS system were characterized by greater sensitivity than those performed with DAD. In fact, LOQs measured with MS were in the range 18–170 ng mL^−1^ while those obtained with DAD ranged from 50 to 500 ng mL^−1^ for all analytes. For this reason, HPLC-MS was chosen for quantification. Repeatability was measured on the same day (intraday repeatability) and on different days (interday repeatability) by injecting three different concentrations of standard mixtures five times on the same day and on three consecutive days. The intraday and interday repeatability was from 1.5 to 5.1% and from 4.2 to 9.7%, respectively.

### 2.2. Phytochemical Characterization of the Extracts

#### 2.2.1. Total Phenolic and Flavonoid Content

**Figure 1:**
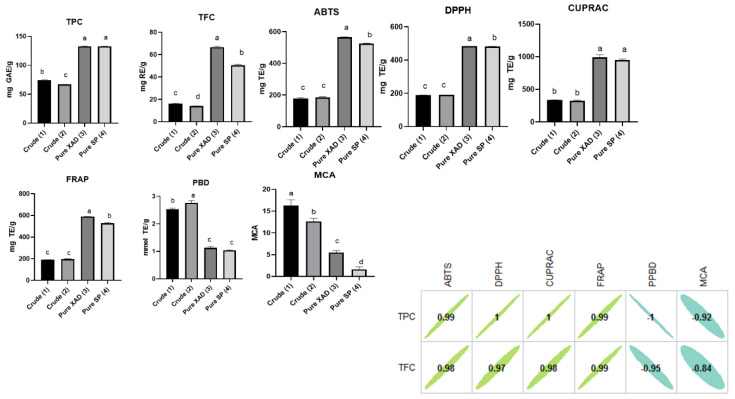
Total phenolic (TPC), flavonoid (TFC), and antioxidant properties and Pearson’s correlation between total bioactive compounds and antioxidant properties (PPBD: Phosphomolybdenum; MCA: Metal chelating activity). GAE: Gallic acid equivalent; RE: Rutin equivalent; TE: Trolox equivalent; EDTAE: EDTA equivalent. Values expressed are means ± S.D. of three parallel measurements. Different letters indicate significant differences in the extracts (*p* < 0.05).

Total phenolic and flavonoid content of extracts can serve as a good indicator of their biological potential. Thus, as a first step of characterization of *L. cardiaca* extracts, they were determined using colorimetric methods. The highest level of these compounds was detected in Pure SP (4) and Pure XAD (3) samples, which were two purified extracts using the resins SP207 and XAD7HP, respectively. On the other hand, the crude extract Crude (2) contained the lowest levels of the analyzed compounds (Figure 1 and Table S1). 

The use of spectrophotometric methods is not exhaustive in determining accurate levels of phenolics. For example, in the Folin–Ciocalteu method, the reagent could be reduced by not only phenolics but also non-phenolics (peptides etc.), and thus the obtained results might not be reliable [24,25]. In this sense, advanced chromatographic techniques are helpful to provide certain information regarding the chemical profiles of plant extracts. To this end, the chemical compositions of tested *L. cardiaca* extracts were determined by HPLC-DAD-MS analysis. Data regarding *L. cardiaca* total bioactive compounds levels have been addressed in earlier studies [6,26,27,28,29]. Different levels were observed in these studies. For example, Ebrahimzadeh et al. [27] have reported that the level of total phenolics was 54.3 mg GAE/g extract, which was lower than values in the presented results. In addition, the total phenolic content of *L. cardiaca* was found to be 2.8 mg GAE/g extract in one earlier paper conducted by Armatu et al. [28]. However, in another study [29], the total phenolic level was higher (200 mg GAE/g extract) than values reported in the present study. The different obtained results could be explained by variance in geographical and climatical conditions as well as the different solvents and plant parts used.

#### 2.2.2. Liquid Chromatography–Mass Spectrometry Analysis

The phytochemical analysis of crude and purified *L. cardiaca* extracts led to the quantification of several compounds of biological significance. Table 2 shows the content of the twelve monitored bioactive compounds in four different extracts. 

The values of the total phytochemicals identified were quite similar for the Crude (1) and Crude (2) extracts (28,826.1 ± 1157.0 and 29,288.0 ± 865.2 µg g^−1^, respectively). In the purified extracts, their concentrations increased significantly. Indeed, in the Pure XAD (3) extract which was obtained using the XAD7HP resin, a total content of 45,329.1 ± 886.7 µG g^−1^ of the investigated compounds was detected, while the highest value was found in the Pure SP (4) extract obtained using the SP207 resin (61,252.7 ± 829.1 µg g^−1^). As already described, the higher amounts of total phytochemicals found in these two extracts could be due to the purification step. 

Sepabeads™ SP207 and Amberlite™ XAD7HP are polymeric adsorbent resins supplied as white insoluble beads. Amberlite XAD7HP is a non-ionic aliphatic acrylic polymer, whereas Sepabeads SP207 is a brominated styrenic polymer. Polymeric adsorbents can be described as highly porous structures whose internal surfaces can adsorb and then desorb a wide variety of different species. In polar solvents such as water, they exhibit non-polar or hydrophobic behavior and so can adsorb organic species. The hydrophobicity is most pronounced with the styrenic adsorbents. The adsorption of a particular species can also depend upon its similarity to a particular polymeric adsorbent on the basis that “like attracts like”. The principal parameters for the choice of resins are the nature of the solvent, the functionality of the solute, and the size of the solute. After adsorption, a solute can be desorbed from the resin by changing the solvent so that the new solvent has a higher affinity for the polymer matrix. For example, if a solute was originally adsorbed from a purely aqueous medium, then the use of a solvent, such as acetone or ethanol, could disrupt the solute–resin interaction by better solvating the solute. In some cases, a solvent/aqueous mixture may be sufficient to remove the solute and the use of a gradient can give selective desorption of certain species. In our study, the XAD7HP resin was shown to be less able to retain phenolic acids when compared with the SP207 one. Different classes of compounds were monitored in the studied samples, such as phenolic acids (chlorogenic, caffeic, caffeoylmalic, and *trans*-ferulic acids), flavonoids (rutin and quercetin), phenylethanoid glycosides (verbascoside and lavandulifolioside), iridoids (harpagide), guanidine pseudoalkaloids (leonurine), triterpenes (ursolic aicd), and labdane diterpenes (forskolin). Among the four extracts, the Pure SP (4) extract showed the major content of harpagide, leonurine, and forskolin (3748.0 ± 113.6, 523.3 ± 12.4, 28.5 ± 1.8 µg g^−1^, respectively), whereas the Crude (2) extract showed the major content of ursolic acid (10.5 ± 0.3 µg g^−1^). These compounds showed relevant biological activities. For instance, a recent study showed that leonurine acts on the nervous system, increasing the cell area, total neurite length, and maximum neurite length of corticosterone-induced PC12 cells [30]. Instead, concerning the phenolic acids, the highest values were found in the Pure SP (4) extract (33,987.9 ± 880.2 µg g^−1^), while lower values were detected in the Pure XAD (3) extract (10,505.5 ± 458.4 µg g^−1^). Among phenolic compounds, caffeoylmalic acid was found to be the main constituent (average concentration in the four extracts of 10,143.6 µg g^−1^), followed by chlorogenic acid (average concentration of 3838.1 µg g^−1^), *trans*-ferulic acid (average concentration of 2719.8 µg g^−1^), and caffeic acid (average concentration of 1625.7 µg g^−1^). The values of phenolic compounds were higher in the purified extracts when compared with the crude extracts, and this is in accordance with the data reported by Flemming et al., who also reported higher values for this class of compounds in the refined aqueous extract of *L. cardiaca* with respect to the ethanolic extract [31]. On the other hand, the highest flavonoid content was found in the Pure XAD (3) extract (6905.6 ± 150.6 µG g^−1^), whereas the lowest one was found in the Crude (2) extract (2435.0 ± 3.15 µg g^−1^). Rutin was more abundant than quercetin, with an average concentration of 3967.1 µg g^−1^. The content of rutin was higher in Pure XAD (3) with respect to the crude extracts and these values are in accordance with those reported in the literature [31]. These flavonoids are listed in the current European Pharmacopoeia (Ph. Eur.), being endowed with noteworthy pharmacological activities [32]. Phenylethanoid glycosides are another important class of compounds in terms of bioactivity. For instance, lavandulifolioside is able to produce a significant negative chronotropism, prolongation of the atrioventricular (P-Q and Q-T) intervals, and a decrease in blood pressure [8]. Concerning our study, the highest content of phenylethanoid glycosides was detected in the Pure XAD (3) extract obtained with the XAD7HP resin (27,552.1 ± 261.7 µg g^−1^), while the lowest amounts were found in the Crude (1) extract (10,870.6 ± 122.1 µg g^−1^). In particular, verbascoside was found at higher concentrations than lavandulifolioside (average concentration of 10,220.9 and 6871.6 µg g^−1^, respectively). Plants rich in verbascoside have been used in folk medicine to treat inflammation and microbial infections for several years. Over the course of many years, numerous scientific investigations have demonstrated its beneficial activities for human health, including antioxidant, anti-inflammatory, wound-healing, neuroprotective, and antineoplastic properties [19]. Finally, from a comparison with literature data, the percentage concentrations of chlorogenic acid, caffeic acid, ferulic acid, verbascoside, and rutin found in our study were higher than the values reported by Ritter et al. [4], probably due to different extraction procedures but also to different plant variability, e.g., plant origin, season of harvesting, and plant maturation. 

### 2.3. Antioxidant Properties

Several studies indicated that oxidative stress is considered as the main contributor to the progression of chronic and degenerative diseases. The knowledge on this process has originated the term “oxidative stress-related diseases” in the last decade. Oxidative stress-related diseases include cardiovascular disease, diabetes, or Alzheimer’s disease, which are among the world’s biggest killers [33]. From this point, we need to control the balance between the production of free radicals and the antioxidant defense system. Thus, dietary antioxidant compounds have great importance for the control mechanisms [34,35]. In light of this information, we examined the antioxidant properties of *L. cardiaca* extracts via different chemical assays. Among these assays, DPPH and ABTS were used to detect the removal capacity of free radicals by plant extracts. In both assays, the scavenging abilities of *L. cardiaca* extracts can be ranked as Pure XAD (3) > Pure SP (4) > Crude (2) > Crude (1) (Figure 1 and Table S2). Reducing power assays, namely CUPRAC and FRAP, reflect electron-donating abilities of antioxidant compounds. By electron-donating, the metal ions are reduced (Cu^2+^ to Cu^+^ in CUPRAC assay; Fe^3+^ to Fe^2+^ in FRAP assay). The highest values in the assays were observed for the purified extracts Pure XAD (3) and Pure SP (4). The strongest extracts in both free radicals and reducing power assays were those which contained the highest level of total phenolics. This is based on the fact that phenolic compounds are the main contributors to the antioxidant properties of the tested extracts. This assumption was also confirmed by correlation analysis, where we observed a linear correlation between total phenolics and the above-mentioned assays (Figure 1). In addition, many authors reported a strong correlation between total phenolics and antioxidant properties [36,37]. As another antioxidant mechanism, chelation reflects the neutralization of transition metal and this could stop the production of the hydroxyl radical, which is the most dangerous radical. In the current paper, the strongest chelating ability was provided by the crude extracts Crude (1) and Crude (2), followed by the purified ones, Pure XAD (3) and Pure SP (4). Since non-phenolics such as polysaccharides and peptides have chelation ability as well as phenolics, contradictory results were observed in the metal chelation assay. However, some researchers found a low correlation between them. The phosphomolybdenum assay is one of the total antioxidant assays. In fact, the assay could be considered one of the reducing power assays (based on the transformation of Mo (VI) to Mo (V)). However, in addition to phenolics, other antioxidants (terpenoids, vitamin C, tocopherols, etc.) could play a role in the assay. In contrast to other antioxidant assays, crude extract Crude (2) exhibited the best ability, followed by the crude extract Crude (1), and the purified extracts Pure XAD (3) and Pure SP (4). In accordance with our findings, a weak relationship between total phenolic levels and phosphomolybdenum abilities was found. According to literature data, several studies were performed on the antioxidant properties of *L. cardiaca* [27,28]. Since the authors used different expression methods, such as IC_50_ and ascorbic acid equivalents, a comparison with the presented results was almost impossible. Taken together, our presented results have been proven to offer good support for the potential use of *L. cardiaca* in the food and pharmaceutical industries.

### 2.4. Enzyme Inhibitory Properties

In the last century, humanity has been faced with several health problems. These are known as global health problems and their morbidities and mortalities are increasing day by day. For example, 50 million people are living with Alzheimer’s diseases and almost 10 million new cases are expected every year [38]. As another example, 422 million people have diabetes worldwide [39]. At this point, we need to answer the question, “how can we control the progression of these diseases?” Thus, we need effective weapons against diseases. Although several therapeutic strategies have been reported for this purpose, most of them are not enough to control diseases. It has been shown that enzymes play a pivotal role in the development of several diseases. The inhibition of enzymes could alleviate the symptoms in the pathologies of the diseases. For instance, cholinesterases hydrolyze acetylcholine in the synaptic gap, and this stops neural transmission. In this sense, if cholinesterase is inhibited, the neural transmission could be continued, and thus cognitive functions could be improved in Alzheimer’s patients [40]. Regarding the connection of diabetes mellitus and enzymes, amylase and glucosidase are the main enzymes for hydrolyzing carbohydrates. The inhibition of amylase and glucosidase actions could be useful to manage the blood glucose level in diabetes mellitus patients after a carbohydrate-rich diet [41]. As another example, tyrosinase plays a pivotal role in the synthesis of melanin and thus the inhibition of tyrosinase could control the hyperpigmentation problems linked to a high level of melanin [42]. For this reason, these enzymes (cholinesterases, amylase, glucosidase, tyrosinase, lipase, etc.) are considered pharmaceutical target boards [15,16]. In the pharmaceutical industries, several compounds have been produced as enzyme inhibitors, but most of them displayed unpleasant side effects [43,44]. In this sense, new raw materials from natural sources are needed as a source of enzyme inhibitors. 

**Figure 2:**
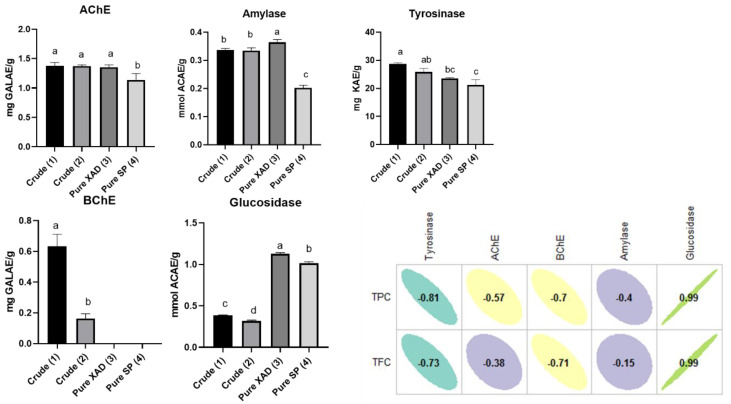
Enzyme inhibitory properties and Pearson’s correlation between total bioactive compounds and enzyme inhibitory properties. Values expressed are means ± S.D. of three parallel measurements. GALAE: Galatamine equivalent; KAE: Kojic acid equivalent; ACAE: Acarbose equivalent. Ni: no inhibition. Different letters indicate significant differences in the extracts (*p* < 0.05).

In the present study, to detect inhibitory activities, the *L. cardiaca* extracts were tested against cholinesterases, tyrosinase, amylase, and glucosidase. The results are given in Figure 2. 

The acetylcholinesterase (AChE) inhibition ability of three extracts (Crude (1), Crude (2) and Pure XAD (3) was very similar (1.35–1.38 mg GALAE/g) and the lowest inhibition was observed for the extract Pure SP (4) obtained by the SP207 resin. However, galantamine exhibited the best AChE inhibitory ability, with the lowest IC_50_ value (0.003 mg/mL). (Table S3). Regarding butyrylcholinesterase (BChE) inhibition ability, two samples (Pure XAD (3) and Pure SP (4)) were not active, and the best ability was recorded for the crude extract Crude (1). The best tyrosinase ability was detected for the crude extracts Crude (1) and Crude (2), followed by the purified extracts Pure XAD (3) and Pure SP (4). When compared with extracts, kojic acid had an excellent inhibitory effect on tyrosinase (IC_50_: 0.09 mg/mL). On the other hand, the extract Pure XAD (3) purified by XAD7HP exhibited the strongest amylase and glucosidase inhibition abilities (Table S3). Acarbose displayed stronger amylase (IC_50_: 0.80 mG/mL) and glucosidase (IC_50_: 0.86 mG/mL) effects when compared with all evaluated extracts. Some compounds in the chemical composition of *L. cardiaca* extracts have been reported as significant enzyme inhibitors. For example, verbascoside [19], rutin [45], quercetin [46], and ursolic acid [47] exhibited good enzyme inhibitory potential in earlier studies. From this point, *L. cardiaca* could be considered a valuable source of enzyme inhibitor agents. However, further studies on the enzyme inhibitory abilities of purified compounds from *L. cardiaca* could be suggested for determining responsible compounds in the performed assays as well as enzyme inhibition types. Thus, the presented results could provide a good basis for exploring the potential of *L. cardiaca.*

### 2.5. Relationship between Bioactivities and Chemical Composition

**Figure 3:**
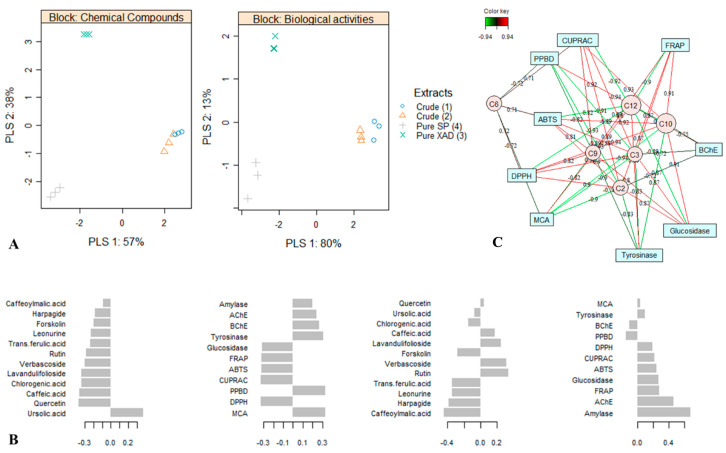
Partial least squared analysis. (**A**) Biplot describing the phytochemical and bioactivity variation between the extracts. (**B**) Loadings plot of each variable selected on the first two functions in each data matrix. (**C**) Network displaying the correlation between the bioactivities and phytochemical compounds. C2 = chlorogenic acid, C3 = caffeic acid, C6 = verbascoside, C9 = lavandulifolioside, C10 = quercetin, C12 = ursolic acid.

As the next step, the Partial Least Square (PLS) model was performed with the phytochemical compounds and biological activities datasets. Partial least square analysis, belonging to the traditional multiple regression models, is a highly efficient model to visualize possible relationships between the molecules and biological activities [48]. From the PLS biplot shown in Figure 3A, it can be seen that the samples of *L. cardiaca* were well discriminated into three groups. 

In both blocks, the crude extracts Crude (1) and Crude (2), being close to each other, were located on the positive quadrant of PLS1, with a variation of 57 and 80%, respectively. In addition, the purified extracts Pure XAD (3) and Pure SP (4), being in the negative quadrant of PLS1, were separated along PLS2, with a variance of 38 and 13%, respectively. Based on the loadings plot (Figure 3B), in the first block, PLS1 was positively associated with ursolic acid and negatively with quercetin, caffeic acid, lavandulifolioside, verbascoside, chlorogenic acid, and rutin. In addition, PLS2 was positively linked to rutin and verbascoside and negatively to caffeoylmalic acid, harpagide, leonurine, and *trans*-ferulic acid. Concerning the second block, PLS1 had a high positive loading for Metal Chelating Activity (MCA), phosphomolybdenum (PPBD), tyrosinase, and BChE and strong negative loading for DPPH, CUPRAC, ABTS, FRAP, and glucosidase. Similarly, PLS2 was predominantly correlated to amylase and AChE. Therefore, it can be suggested that the higher MCA, PPBD, anti-tyrosinase, and anti-glucosidase activities of the crude extracts Crude (1) and Crude (2) might be due to their high content of ursolic acid, whereas the stronger antioxidant and anti-glucosidase activity of the purified extracts Pure SP (4) and Pure XAD (3) was due to their greater content of quercetin, caffeic acid, lavandulifolioside, verbascoside, chlorogenic acid, and rutin. This suggestion was reinforced by the elucidation of the correlation between the biological activities and phytochemical compositions, as reported in Figure 3C. As observed, the anti-tyrosinase, MCA, PPBD, anti-BChE activities were positively correlated with the content of ursolic acid, in all r > 0.8. Similarly, quercetin, caffeic acid, lavandulifolioside, verbascoside, and chlorogenic acid were positively linked to the antioxidant and anti-glucosidase activities, overall r > 0.8. Thus, these metabolites influenced the variation in the majority in the bioactivities found in *L. cardiaca* extracts.

## 3. Materials and Methods

### 3.1. Chemicals and Reagents

Caffeic acid (analytical standard, ≥98%, C_9_H_8_O_4_, molecular weight 180.16, Cas No 331-39-5), caffeoylmalic acid (analytical standard, C_13_H_12_O_8_, molecular weight 296.23, Cas No 39015-77-5), chlorogenic acid (analytical standard, ≥95% (titration), C_16_H_18_O_9_, molecular weight 354.31, Cas No 327-97-9), forskolin (analytical standard, C_22_H_34_O_7_, molecular weight 410.50, Cas No 66575-29-9), harpagide (analytical standard, C_15_H_24_O_10_, molecular weight 364.35, Cas No 6926-08-5), leonurine (analytical standard, ≥98% (HPLC), C_14_H_21_N_3_O_5_, molecular weight 311.33, Cas No 24697-74-3), quercetin (analytical standard, ≥95% (HPLC), C_15_H_10_O_7_, molecular weight 302.24, Cas No 117-39-5), rutin (analytical standard, C_27_H_30_O_16_, molecular weight 610.52, Cas No 153-18-4), trans-ferulic acid (analytical standard, 99%, C_10_H_10_O_4_, molecular weight 194.18, Cas No 537-98-4), ursolic acid (analytical standard, purity, C_30_H_48_O_3_, molecular weight 456.70, Cas No 77-52-1), and verbascoside (analytical standard, ≥99% (HPLC), C_29_H_36_O_15_, molecular weight 624.59, Cas No 61276-17-3) were purchased from Sigma Aldrich (St. Louis, MO, USA). Individual stock solutions of each analyte, at a concentration of 1000 µg mL^−1^, were prepared by dissolving pure standard compounds in HPLC-grade methanol and storing them in glass-stoppered bottles at 4 °C, with the exception of leonurine, which was prepared at concentrations of 500 µg mL^−1^. Afterwards, standard working solutions at various concentrations were prepared daily by appropriate dilution of the stock solutions with methanol. HPLC-grade methanol was supplied by Carlo Erba (Milan, Italy). HPLC-grade formic acid (99%) was obtained from Merck (Darmstadt, Germany). All other chemicals were analytical grade. Deionized water was further purified using a Milli-Q SP Reagent Water System (Millipore, Bedford, MA, USA). Before HPLC analysis, all solvents and solutions were filtered through a 0.2-µM polyamide filter from Sartorius Stedim (Goettingen, Germany) and all samples were filtered with Phenex™ RC 4 mm 0.2 µM syringeless filter, Phenomenex (Castel Maggiore, Italy). The resin Amberlite^TM^ XAD7HP was purchased from Sigma Aldrich (St. Louis, MO, USA), while the resin Sepabeads^TM^ SP207 was purchased from Pyvot Tech (New York, NY, USA). 

### 3.2. Plant Materials and Extract Preparation

*L. cardiaca* aerial parts were harvested in Poland in 2020 between the end of April and the beginning of May. For the study, four kind of extracts were prepared. Two of them were crude extracts, differentiating the extraction temperature such as 25 °C (Crude (1)) and 60 °C (Crude (2)). The other two extracts, originating from the *L. cardiaca* aerial parts at 60 °C, were obtained through different adsorption resins, namely XAD7-HP (Pure XAD) and SP207 (Pure SP). The extracts were prepared as described below. The crude extract Crude (1) was prepared from the aerial parts of *L. cardiaca* (5.1 g), which were milled through a 1-mm grid and extracted with 150 mL of 70% ethanol (*v*/*v*) at 25 °C overnight. The hydroalcoholic solution was filtered and concentrated to dryness to give 1.3 g of total extract (yield 25.5% *w*/*w*). For the crude extract Crude (2), *L. cardiaca* aerial parts (40 g) were milled through a 6-mm grid and then extracted twice with 600 mL of 70% ethanol (*v*/*v*) for 3 h at 60 °C. The hydroalcoholic extracts were filtered, combined, and concentrated to dryness to give 8.9 g of total extract (yield 21.7% *w*/*w*). The purified extract Pure XAD (3) was prepared from *L. cardiaca* aerial parts (40 g), which were milled through a 6-mm grid and extracted twice with 600 mL of 70% ethanol (*v*/*v*) for 3 h at 60 °C. The choice of the solvent, restricted to ethanol/water mixtures, is due to the fact that our purpose was the preparation of extracts suitable as food ingredients. The extraction conditions (70% EtOH, 60 °C) were chosen after an initial screening carried out by (thin layer chromatography) TLC and evaluating extract yields. The extraction with 70% ethanol, room temperature (25 °C), was carried out in order to reproduce the conditions described in EMA monography (EMA/HMPC/127430/2010) for the preparation of the tincture (traditional extract) [5]. The hydroalcoholic extracts were filtered, combined, and concentrated in order to obtain an aqueous solution. The latter was clarified by centrifugation and loaded onto a column of Amberlite^TM^ XAD7HP (approximately 100 mL). The initial aqueous eluate was discarded. The column was washed with 100 mL of water and then it was eluted with 70% ethanol (300 mL). The hydroalcoholic eluate was concentrated to dryness and further dried at 50 °C under vacuum to give 1.4 g of purified extract (yield 3.5% *w*/*w*). For the preparation of the purified extract Pure SP (4), *L. cardiaca* aerial parts (40 g) were milled through a 6-mm grid and extracted twice with 600 mL of 70% ethanol (*v*/*v*) for 3 h at 60 °C. The hydroalcoholic extracts were filtered, combined, and concentrated in order to obtain an aqueous solution. The latter was clarified by centrifugation and loaded onto a column of Sepabeads^TM^ SP207 (approximately 100 mL). The initial aqueous eluate was discarded, and the column was washed with 100 mL of water. Afterwards, it was eluted with 70% ethanol (300 mL). The hydroalcoholic eluate was concentrated to dryness and further dried at 50 °C under vacuum to give 2.3 g of purified extract (yield 5.8% *w*/*w*).

### 3.3. HPLC-DAD-MS Analysis

Each extract was dissolved in MeOH (2 mg in 1 mL) and sonicated for 10 min at room temperature; before liquid chromatography–diode array detector–mass spectrometry (LC-DAD-MS) analysis, it was filtered with a 0.2-µM filter. The quantification of bioactive compounds was carried out using a 1290 Infinity series liquid chromatograph (Agilent) with a diode array detector (DAD) and a 6420 Triple Quadrupole (Agilent) equipped with an electrospray ionization (ESI) source operating in negative ionization mode. The separation of target compounds was achieved on a Synergi Polar-RP 80 A (250 mm × 4.6 mm i.d., 4 µm) analytical column (Phenomenex, Torrance, CA, USA). The mobile phase was a mixture of water (A) and methanol (B), both with formic acid 0.1%, and the separation of the monitored compounds was performed at a flow rate of 0.8 mL min^−1^ in gradient elution mode. The composition of the mobile phase varied as follows: 0–5 min, isocratic condition, 40% B; 5–15 min, 95% B; 15–25 min, isocratic condition, 95% B; 25–37 min, 40% B; 37–45 min, 40% B. The injection volume was 5 µL. The temperature of the column was 30 °C and the temperature of the drying gas in the ionization source was 350 °C. The gas flow was 12 L/min, the nebulizer pressure was 60 psi, and the capillary voltage was 4000 V. Detection was performed in the “selected ion monitoring” (SIM) mode and the peak areas of the selected ions were integrated for quantification. The most abundant ions were used for quantitation, and the other for qualification. Analytes were also confirmed by monitoring them at different wavelengths, i.e., 325 nm for chlorogenic, caffeic, caffeoylmalic and trans-ferulic acid, verbascoside, and lavandulifolioside; 210 nm for harpagide, forskolin, and ursolic acid; 280 nm for leonurine, and 256 for rutin and quercetin. The selected ions, the mass spectrometer parameters, and the monitored wavelengths are reported in Table 3.

### 3.4. Total Phenolic and Flavonoid Content 

The total phenolic and flavonoid contents of the extracts were measured as described previously [49]. Standards, namely gallic acid (GAE) for phenolics and rutin (RE) for flavonoids, were used to explain the results.

### 3.5. Antioxidant and Enzyme Inhibitory Properties

The antioxidant potential of the extracts/compound was tested using a series of assays which included phosphomolybdenum, antiradical (DPPH and ABTS), reducing power (FRAP and CUPRAC), and also ferrous chelating assays, following the methods published by Grochowski et al. [50]. Trolox equivalents were used for the expression of antioxidant activities (phosphomolybdenum, DPPH, FRAP, and CUPRAC assays). Disodium edetate (EDTA) was the reference molecule for the metal chelating assay. All tests were conducted in triplicate and results are expressed as mg Trolox per gram of dried extract (TEs/g extract), with the exception of those obtained with phosphomolybdenum assay, expressed in mmol TEs/g. The key enzyme inhibition activity of extracts against acetylcholinesterase (ACh, EC3.1.1.7), butyrylcholinesterase (BuCh, EC 3.1.1.8), tyrosinase (1.14.18.1), α-glucosidase (EC 3.2.1.20), and α-amylase (EC 3.2.1.1) was measured using the protocols published by Grochowski et al. [50]. Galanthamine (1–5 µg/mL) was used as an inhibitor for AChE and BuCh, kojic acid (0.2–1 mM/mL) for tyrosinase, and acarbose (0.2–1 mG/mL) for α-glucosidase and α-amylase activity assays. To provide comparison with inhibitors, IC_50_ values were also given (this is the extract concentration required for inhibiting 50% of the enzyme in the inhibitory assays). All experiments were conducted in triplicate and results are expressed as mg standard equivalent per gram of dry extract.

### 3.6. Statistical Analysis 

All quantitative analyses were performed in triplicate (*n* = 3) and data were expressed as means ± S.D. The bioactivities were determined in tree replicates. R software (Version 3.6.2) was used for the statistical analysis. Significant differences between the extracts were evaluated by analysis of variance (ANOVA), with a probability value of 5%. Pearson’s correlation was estimated to identify the relationship between TPC, TFC, and the biological activities. Then, partial least square analysis was done to determine the contribution of the phytochemical compounds to the biological activities. 

## 4. Conclusions

The present research reported an extensive characterization of *L. cardiaca* extracts and the investigation of their antioxidant and enzyme inhibitory properties. We developed a purification step during extraction, allowing to increase the level of bioactive compounds. Notably, the Sepabeads SP207 resin allowed us to obtain extracts richer in phenolic acids, such as caffeoylmalic acid, *trans*-ferulic acid and chlorogenic acid, and iridoids such as harpagide, which improved each extract’s inhibitory effects on glucosidase. On the other hand, Amberlite XAD7HP resin determined a higher recovery of flavonoids, namely rutin, and phenylethanoid glycosides such as verbascoside and lavandulifolioside, with increased activity on amylase and glucosidase. Overall, the purified extracts displayed higher levels of polyphenols (TPC) and flavonoids (TFC) as well as superior radical scavenging activities and reducing power. Notably, when compared with the crude extracts, the purified ones were characterized by higher levels of quercetin, caffeic acid, lavandulifolioside, verbascoside, chlorogenic acid, and rutin. Clearly, the variation in the chemical composition of the extracts affected their bioactivities. Results demonstrated that the *L. cardiaca* extracts possess good levels of phytochemicals with interesting antioxidant properties. Moreover, the extracts obtained from this plant could be considered valuable sources of enzyme inhibitory agents. Indeed, the purified extracts of *L. cardiaca*, when incorporated in food supplements, could be potentially useful to manage the blood glucose level in diabetes mellitus patients after a carbohydrate-rich diet. Therefore, it is hoped that this research will lay the foundation for exploring the potentiality of *L. cardiaca* to develop, in the future, nutraceuticals and supplement products.

## Figures and Tables

**Table 1:** High-Performance Liquid Chromatography coupled to Diode Array Detector and Mass Spectrometry (HPLC-DAD-MS) validation parameters, i.e., determination coefficient (R^2^), limit of detection (LOD), limit of quantification (LOQ), and repeatability.

Compounds	HPLC-DAD			HPLC-MS			Repeatability ^2^	
	R^2^	LOD (µg mL^−1^)	LOQ (µg mL^−1^)	R^2^	LOD (µg mL^−1^)	LOQ (µg mL^−1^)	Interday	Intraday
Chlorogenic acid	0.9961	0.043	0.130	0.9997	0.017	0.050	6.8	2.1
Caffeic acid	0.9952	0.033	0.100	0.9908	0.017	0.050	7.3	3.4
Caffeoylmalic acid	0.9991	0.105	0.315	0.9997	0.050	0.150	6.5	2.2
*trans*-Ferulic acid	0.9979	0.017	0.050	0.9995	0.006	0.018	4.9	4.0
Leonurine	0.9981	0.167	0.500	0.9988	0.017	0.050	7.4	3.9
Verbascoside	0.9995	0.050	0.150	0.9948	0.017	0.050	4.2	3.6
Rutin	0.9975	0.167	0.500	0.9974	0.010	0.030	5.6	5.1
Quercetin	0.9956	0.167	0.500	0.9918	0.017	0.050	8.5	5.1
Harpagide	n.d. ^1^	n.d.	n.d.	0.9996	0.028	0.085	5.6	4.2
Forskolin	n.d.	n.d.	n.d.	0.9937	0.033	0.100	9.7	3.7
Ursolic acid	n.d.	n.d.	n.d.	0.9929	0.057	0.170	6.3	1.5

^1^ n.d., not determined. ^2^ repeatability has been reported only for HPLC-MS.

**Table 2:** Phytochemical content, expressed as µg g^−1^, in different extracts of *L. cardiaca*.

Compound	*L. Cardiaca* Extracts			
	Crude (1)	Crude (2)	Pure XAD (3)	Pure SP (4)
Chlorogenic acid	2194.4 ± 169.2	2778.9 ± 74.7	4012.1 ± 184.2	6367.1 ± 108.2
Caffeic acid	719.2 ± 23.3	791.3 ± 31.9	2704.7 ± 85	2287.8 ± 34.4
Caffeoylmalic acid	9799.1 ± 832.0	9488.0 ± 227.5	2783.1 ± 134.8	18,504.1 ± 598.3
*trans*-Ferulic acid	1455.9 ± 225.3	1588.9 ± 75.7	1005.5 ± 54.4	6829.0 ± 139.3
Leonurine	111.6 ± 19.7	115. 5 ± 5.8	70.7 ± 3.9	523.3 ± 12.4
Lavandulifolioside	4474.2 ± 75.0	4529.8 ± 150	10,443.2 ± 10.4	8039.1 ± 140.8
Verbascoside	6396.5 ± 47.2	6467.5 ± 245	17,108.9 ± 251,3	10,911.1 ± 92.8
Rutin	2552.8 ± 13.3	2426.4 ± 3.6	6892.4 ± 150.3	3996.8 ± 54.6
Quercetin	9.9 ± 0.9	8.6 ± 0.5	13.2 ± 0.3	13.8 ± 0.8
Harpagide	1078.9 ± 70.9	1068.5 ± 56.2	272.5 ± 12.1	3748.0 ± 113.6
Forskolin	23.9 ± 3.4	14.0 ± 0.9	18.0 ± 0.4	28.5 ± 1.8
Ursolic acid	9.8 ± 1.3	10.5 ± 0.3	4.8 ± 0.5	4.2 ± 0.7
Total phenolic acids	14,168.6 ± 1203.1	14,647.1 ± 409.8	10,505.5 ± 458.4	33,987.9 ± 880.2
Total flavonoids	2562.7 ± 12.4	2435.1 ± 3.2	6905.6 ± 150.6	4010.6 ± 55.5
Total phenylethanoid glycosides	10,870.6 ± 122.1	10,997.3 ± 395.4	27,552.1 ± 261.7	18,950.2 ± 233.6
Total phytochemicals	28,826.1 ± 1157.0	29,288.0 ± 865.2	45,329.1 ± 886.7	61,252.7 ± 829.1

**Table 3:** HPLC-DAD-MS parameters for the quantification of 12 bioactive compounds in *L. cardiaca* extracts.

Compound	Time Windows (min)	Wavelength (nm)	Ions (*m*/*z*)	Retention Time (Rt) (min)	Polarity
Harpagide	2.5–6.0	210	409 ^a^, 363	4.4	Negative
Chlorogenic acid	6.0–8.4	325	353 ^a^, 707	7.4	Negative
Caffeic acid	8.4–13.5	325	179	9.4	Negative
Caffeoylmalic acid	8.4–13.5	325	295 ^a^, 591	9.9	Negative
*trans*-Ferulic acid	8.4–13.5	325	193	12.5	Negative
Leonurine	8.4–13.5	280	310	10.5	Negative
Lavandulifolioside	8.4–13.5	325	755	11.0	Negative
Verbascoside	8.4–13.5	325	623	11.3	Negative
Rutin	8.4–13.5	256	609	12.3	Negative
Quercetin	13.5–16.0	256	301	14.8	Negative
Forskolin	16.0–18.0	210	409	17.0	Negative
Ursolic acid	18.0–22.0	210	455	19.7	Negative

^a^ These ions have been used for quantification, the others for confirming the analytes.

## Data Availability

Data available on request.

## Supplementary Materials

The following are available online at https://www.mdpi.com/2223-7747/10/2/195/s1, Table S1: Total bioactive components in the tested extracts, Table S2: Antioxidant properties of the tested extracts, Table S3: Enzyme inhibitory properties of the tested extracts.
