# Supplementary material for: Phytochemical Profile and Biological Activities of Crude and Purified Leonurus cardiaca Extracts
Source: Plants (Basel). 2021 Jan 21;10(2):195. doi: 10.3390/plants10020195 (PMC7911824; doi:10.3390/plants10020195)
Supplement: Supplementary file 1 [file plants-10-00195-s001.pdf]

Table S1. Total bioactive components in the tested extracts*.

Extracts	Total phenolic content (mg GAE/g)	Total flavonoid content (mg RE/g)
Crude (1)	74.48±0.88^{b}	16.25±0.32^{c}
Crude (2)	66.90±0.17^{c}	14.05±0.11^{d}
Pure XAD (3)	132.40±1.07^{a}	66.69±0.65^{a}
Pure SP (4)	132.73±0.33^{a}	50.54±0.68^{b}

* Values expressed are means ± S.D. of three parallel measurements. GAE: Gallic acid equivalent; RE: Rutin equivalent. Different letters indicate significant differences in the extracts (p< 0.05).

Table S2. Antioxidant properties of the tested extracts*.

Extracts	DPPH (mg TE/g)	ABTS (mg TE/g)	CUPRAC (mg TE/g)	FRAP (mg TE/g)	Chelating ability (mg EDTAE/g)	Phosphomolybdenum (mmol TE/g)
Crude (1)	189.45±0.66^{c}	179.25±4.75^{c}	338.84±0.75^{b}	190.70±0.43^{c}	16.30±1.32^{a}	2.53±0.05^{b}
Crude (2)	190.68±0.20^{c}	185.74±4.09^{c}	327.24±6.29^{b}	197.01±3.03^{c}	12.61±0.76^{b}	2.77±0.08^{a}
Pure XAD (3)	483.86±0.12^{a}	564.91±3.56^{a}	991.90±38.34^{a}	588.79±1.31^{a}	5.55±0.43^{c}	1.13±0.05^{c}
Pure SP (4)	481.96±0.84^{b}	525.45±2.33^{b}	953.38±14.97^{a}	528.83±4.35^{b}	1.61±0.61^{d}	1.04±0.01^{c}

* Values expressed are means ± S.D. of three parallel measurements. TE: Trolox equivalent; EDTAE: EDTA equivalent. Different letters indicate significant differences in the extracts (p< 0.05).

Table S3. Enzyme inhibitory properties of the tested extracts*.

Extracts	AChE inhibition		BChE inhibition		Tyrosinase inhibition		Amylase inhibition		Glucosidase inhibition	
	mg GALAE/g	IC_{50} (mg /ml)	mg GALAE/g	IC_{50} (mg /ml)	mg KAE/g	IC_{50} (mg /ml)	mmol ACAE/g	IC_{50} (mg /ml)	mmol ACAE/g	IC_{50} (mg /ml)
Crude (1)	1.38±0.06[a]	1.96±0.06[b]	0.63±0.08[a]	>5	28.72±0.33[a]	3.11±0.04[b]	0.34±0.01[b]	3.67±0.04[c]	0.39±0.01[c]	3.47±0.07[d]
Crude (2)	1.37±0.02[a]	1.97±0.03[b]	0.16±0.03[b]	>5	25.85±1.27[ab]	3.46±0.17[bc]	0.34±0.01[b]	3.70±0.08[c]	0.31±0.01[d]	4.27±0.16[e]
Pure XAD (3)	1.35±0.04[a]	2.00±0.06[b]	Ni	Ni	23.55±0.26[bc]	3.80±0.04[cd]	0.37±0.01[a]	3.38±0.08[b]	1.13±0.01[a]	1.19±0.01[b]
Pure SP (4)	1.14±0.11[b]	2.39±0.24	Ni	Ni	21.25±1.83[c]	4.23±0.35[d]	0.20±0.01[c]	>5	1.02±0.01[b]	1.31±0.02[c]
Galatamine	-	0.003±0.001[a]	-	0.005±0.01	-	Nt	-	Nt	-	Nt
Kojic acid	-	Nt	-	Nt	-	0.09±0.01[a]	-	Nt	-	Nt
Acarbose	-	Nt	-	Nt	-	NT	-	0.80±0.08[a]	-	0.86±0.01[a]

* Values expressed are means ± S.D. of three parallel measurements. GALAE: Galatamine equivalent; KAE: Kojic acid equivalent; ACAE: Acarbose equivalent. Ni: no inhibition. Nt: No tested. Different letters indicate significant differences in the extracts ($p<0.05$, the letter "a" indicates strong ability). IC_{50} (mg/mL), inhibition concentration at which 50% of the enzyme activities were inhibited
